# Immune reconstitution following alemtuzumab therapy is characterized by exhausted T cells, increased regulatory control of proinflammatory T cells and reduced B cell control

**DOI:** 10.3389/fimmu.2023.1249201

**Published:** 2023-09-06

**Authors:** Marina Rode von Essen, Helene Højsgaard Chow, Rikke Holm Hansen, Sophie Buhelt, Finn Sellebjerg

**Affiliations:** Danish Multiple Sclerosis Center, Department of Neurology, Copenhagen University Hospital - Rigshospitalet, Glostrup, Denmark

**Keywords:** alemtuzumab therapy, multiple sclerosis, immune reconstitution, lymphocytes, disease activity, secondary autoimmunity

## Abstract

Alemtuzumab is a monoclonal antibody targeting CD52 on the surface of immune cells, approved for the treatment of active relapsing-remitting multiple sclerosis (RRMS). The purpose of this study was to analyze the repopulation of peripheral lymphocytes following alemtuzumab-induced lymphocyte depletion and investigate associations with disease activity and development of secondary autoimmunity. For this, blood samples were collected two years after initiation of alemtuzumab treatment and lymphocytes were subjected to a comprehensive flow cytometry analysis. Included in the study were 40 patients treated with alemtuzumab and 40 treatment-naïve patients with RRMS. Disease activity and development of secondary autoimmune disease was evaluated after three years of treatment. Our study confirms that alemtuzumab treatment profoundly alters the circulating lymphocyte phenotype and describes a reconstituted immune system characterized by T cell activation/exhaustion, an increased regulatory control of IL-17 producing effector T cells and CD20^+^ T cells, and a reduced control of B cells. There were no obvious associations between immune cell subsets and disease activity or development of secondary autoimmune disease during treatment with alemtuzumab. Our results indicate that the reconstituted immune response is skewed towards a more effective regulatory control of MS-associated proinflammatory T cell responses. Also, the enlarged pool of naïve B cells together with the apparent decrease in control of B cell activity may explain why alemtuzumab-treated patients retain the ability to mount a humoral immune response towards new antigens.

## Introduction

Alemtuzumab is an immune reconstitution therapy approved for the treatment of active relapsing-remitting multiple sclerosis (RRMS) (1). Alemtuzumab effectively reduces the relapse rate and disability worsening in patients with RRMS (2, 3); however, the risk of secondary autoimmune disorders is common with thyroid autoimmune disease being the most frequent (4, 5).

Alemtuzumab is a humanized monoclonal antibody targeting CD52 expressed on the surface of most white blood cells (6). Upon binding, alemtuzumab induces antibody and complement-mediated cytolysis of targeted cells. Cells of the adaptive immune system express the highest level of CD52 and the lowest level of complement inhibitory proteins, and likely as a consequence of this they are the cells most sensitive to alemtuzumab-induced depletion (6). After depletion, the adaptive immune system reconstitutes from precursor cells or mature cells that have escaped depletion. B cells repopulate within 3-6 month reaching or overshooting baseline levels, followed by a delayed T cell reappearance after 1-3 years but with circulating cell counts significantly lower than at baseline (7–9). Furthermore, the reconstitution dynamics of T cell subtypes differ, with regulatory T cells repopulating faster than effector T cells (7, 10). The reconstituted immune system with a higher proportion of regulatory cells has therefore been proposed to contribute to the long-lasting effect of alemtuzumab therapy (10).

Understanding the immunological changes induced by alemtuzumab and its association with clinical outcomes, including development of secondary autoimmunity, is instrumental for the future use of alemtuzumab as an MS treatment. With this study, we therefore thoroughly investigated a wide range of lymphocyte phenotypes in the reconstituted immune system two years after the initiation of alemtuzumab treatment in patients with RRMS and analyzed associations with disease activity and development of secondary autoimmune disease.

## Materials and methods

### Study population

In this observational case-control study, 40 treatment-naïve, newly diagnosed patients with RRMS (29 females/11 males; mean 36 years, range 21-62 years) and 40 patients treated with alemtuzumab (31 females/9 males; mean 39 years, range 22-56 years) at the Danish Multiple Sclerosis Center at Copenhagen University Hospital - Rigshospitalet were included; there were no significant differences in age or sex distribution between groups. The alemtuzumab-treated patients were all included in the recently published national study of Danish patients treated with alemtuzumab (11). The alemtuzumab-treated patients had a mean disease duration of 12 years (range 4-23 years), had a mean EDSS score of 4 (range 2-6.5), and had a mean number of 1.7 relapses (range 0-7) the year prior to the initiation of alemtuzumab therapy. They had received a mean of four previous therapies (range 1-7), and the most recent treatment was fingolimod in 19 patients, natalizumab in 15 and dimethyl fumarate or daclizumab in six. All patients fulfilled the 2017 McDonald criteria for RRMS (12). The alemtuzumab treatment regimen included a first course where 12 mg was infused on five consecutive days. Twelve months later, a second course of 12 mg daily was infused on three consecutive days. Blood samples were obtained from alemtuzumab-treated patients one year later to measure the repopulation of lymphocytes, i.e., two years after initiation of treatment.

### Study protocol approvals, registrations, and patient consent

All participants gave informed, written consent to participation. The study was approved by the regional scientific ethics committee (protocol number H-16047666).

### Disease activity and secondary autoimmunity

The alemtuzumab-treated patients were seen for clinical controls at six-monthly intervals during the first three years after initiation of treatment and underwent magnetic resonance imaging (MRI) scans of the brain before treatment and annually after the initiation of treatment. The occurrence of relapses (defined according to the 2017 McDonald criteria) and secondary autoimmune diseases was based on chart review blinded to the results of the immunological studies. MRI activity was defined as new or enlarging T2 lesions compared to a rebaseline scan obtained one year after the initiation of treatment. No evidence of disease activity (NEDA) was defined as no relapses and no new or enlarging MRI lesions. Confirmed worsening of disability (CDW) was defined as a 1 point increase in Expanded Disability Status Scale (EDSS) score for patients with a baseline EDSS score from 1 to 5 and 0.5 for patients with a baseline EDSS score of 5.5 or more, confirmed after at least six months of follow-up (none of the patients had a baseline EDSS score of 0).

### Flow cytometry analysis of blood

**Figure 1:**
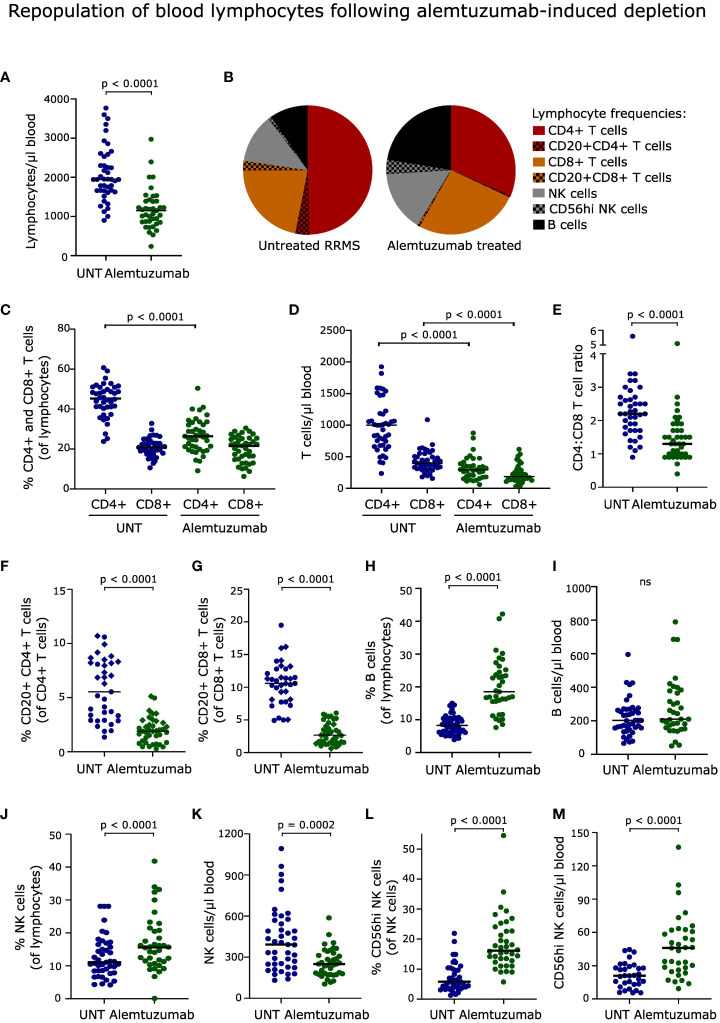
Repopulation of blood lymphocytes. **(A)** Absolute numbers of lymphocytes in the blood of untreated (UNT) and alemtuzumab-treated patients. **(B)** Frequency distribution of lymphocytes. **(C, D)** Frequency and absolute numbers of CD4^+^ and CD8^+^ T cells. **(E)** CD4:CD8 T cell ratio. **(F, G)** Frequency and absolute numbers of CD20^+^CD4^+^ and CD20^+^CD8^+^ T cells. Diamonds and circles represent samples stained with PE/Cy7 and BV421-conjugated anti-CD20 antibodies, respectively. **(H–M)** Frequency and absolute numbers of B cells **(H, I)**, NK cells **(J, K)** and CD56^hi^ NK cells **(L, M)** in untreated (UNT) and alemtuzumab-treated patients. The median value is shown for all groups analyzed. ns, non-significant.

Venous blood was collected, and peripheral blood mononuclear cells (PBMCs) isolated by density gradient centrifugation using Lymphoprep (Axis-Shield, Oslo, Norway) and washed twice in cold PBS/2 mM EDTA within 1 hour of sampling. PBMC were then incubated with FcR blocking reagent (Miltenyi Biotec, Bergisch Gladbach, Germany) to prevent nonspecific Ab binding, and thereafter stained in PBS/2% FBS/0.02% NaN_3_ with a combination of fluorochrome-conjugated antibodies targeting B cells, T cells and NK cells (conjugate; clone): CD3 (APC, APC/Cy7; UCHT1), CD4 (APC/Cy7, PerCP/Cy5.5; OKT4), CD8 (BV605; RPA-T8), CD19 (PerCP/Cy5.5; HIB19), CD25 (PE; M-A251), CD27 (FITC; 323), CD38 (BV421; HIT2), CD45RA (FITC; HI100), CD57 (PB; HNK-1), CD127 (APC; A019D5), CD183 (CXCR3, PE/Cy7; G025H7), CD185 (CXCR5, AF488; J252D4), CD196 (CCR6, BV421; G034E3), CD197 (CCR7, PE; G043H7), CD279 (PD-1, BV605; EH12.2H7) all from BioLegend (CA, USA) and CD56 (PE/Cy7; CMSSB) from eBioscience (ThermoFisher, MA, USA). CD20 on T cells was stained using either CD20-PE/Cy7 or BV421 (2H7, BioLegend) resulting in variability of the CD20-signal. An equal number of untreated and treated patients were stained with each of the two fluorochrome-conjugated antibodies; and depicted in **Figure 1** where diamonds represent CD20-PE/Cy7 and circles CD20-BV421. Isotype matched controls were used to correct for nonspecific Ab binding and spectral overlap, where appropriate. TruCount staining of whole blood to measure absolute cell count was performed using BD Multitest 6-color TBNK Reagent according to manufacturer (BD Biosciences, San Jose, CA, USA). Data were acquired on a FACS Canto II flow cytometer (BD Biosciences) and data analyses performed using the software FlowJo (TreeStar, Ashland, OR, USA). Data analyses were performed blinded.

### Statistical analysis

For analysis of sex differences between groups a Chi-square test was performed, and for analysis of age differences between groups a Mann-Whitney U test was applied. To compare cell frequencies and absolute number of cells between treatment naive and alemtuzumab-treated patients with RRMS a Mann-Whitney U test was performed. To analyze a possible association between immune cells and development of secondary autoimmunity, alemtuzumab-treated patients were divided into a group that developed autoimmune thyroid disease and one that did not and cell frequency, count or ratios of interest assigned to each group; and a Mann-Whitney U test applied. A similar analysis was performed investigating a possible association between immune cells and disease activity. As CD20^+^ T cells were analyzed using two different monoclonal antibodies, CD20^+^ T cells were analyzed in general linear models with the antibody used for analysis as a cofactor. Correlations were assessed by Spearman rank correlation analysis. A significance level of p < 0.005 was considered statistically significant for all analyses. For frequencies percentages are given with 95% confidence intervals (CI).

### STROBE guidelines

For this manuscript the STROBE reporting guidelines for observational studies was used (13).

### Data availability

Data are available in anonymized form and can be shared by request from any qualified investigator. Sharing requires approval of a data transfer agreement in accordance with GDPR and Danish data protection regulation.

## Results

### Disease activity, worsening of disease and secondary autoimmune disease

In the 40 patients treated with alemtuzumab, 16 patients (40%, 95% CI 25-57%) had relapse activity in year one and 21 patients (53%; 95% CI 36-68%) had relapse activity in year two or three after initiation of treatment. The annualized relapse rate during the three years of follow-up was 0.6, i.e., substantially lower than the pre-treatment annualized relapse rate of 1.7. This is consistent with the notion that Danish patients treated with alemtuzumab, including the patients included in the present study, are characterized by having high disease activity and most patients having failed several therapies before initiation of alemtuzumab therapy (11). Twelve out of the 40 patients (30%, 95% CI 17-47%) had new or enlarging T2 lesions on MRI at year two or year three compared to the rebaseline MRI at year one. Thirteen patients (33%, 95% CI 19-49%) had no evidence of disease activity (NEDA) in year two and three.

Six patients (15%, 95% CI 6-30%) had confirmed EDSS worsening at the end of the three years of follow-up. Three of these patients had NEDA during the entire follow-up and three had either relapses or MRI activity during follow-up. The worsening was sustained to the end of follow-up in all patients.

A total of nine out of 40 patients (23%; 95% CI 11-38%) developed autoimmune thyroid disease during the three years of follow-up; none of the patients developed other forms of secondary autoimmune disease during follow-up. Two patients had autoimmune thyroiditis and seven had Grave’s disease and were seropositive for TSH receptor antibodies.

### Repopulation of blood lymphocytes following alemtuzumab-induced depletion

After two years, lymphocyte counts were not fully recovered (p < 0.001, **Figure 1A**), and the distribution of reconstituted lymphocyte populations was profoundly altered in patients treated with alemtuzumab (**Figure 1B**). This included a reduced frequency of CD4^+^ T cells (p < 0.0001, **Figure 1C**) and consequently a decreased CD4:CD8 T cell ratio (p < 0.0001, **Figure 1E**), a reduced frequency of CD20^+^CD4^+^ and CD20^+^CD8^+^ T cells (p < 0.0001, **Figures 1F, G**), an increased frequency of B cells (p < 0.0001, **Figure 1H**) and NK cells (p < 0.0001, **Figure 1J**), including CD56^hi^ NK cells (p < 0.0001, **Figure 1L**), compared to untreated patients. The lower frequency of CD20^+^ T cell subsets was observed regardless of which antibody was used for staining.

**Figure 2:**
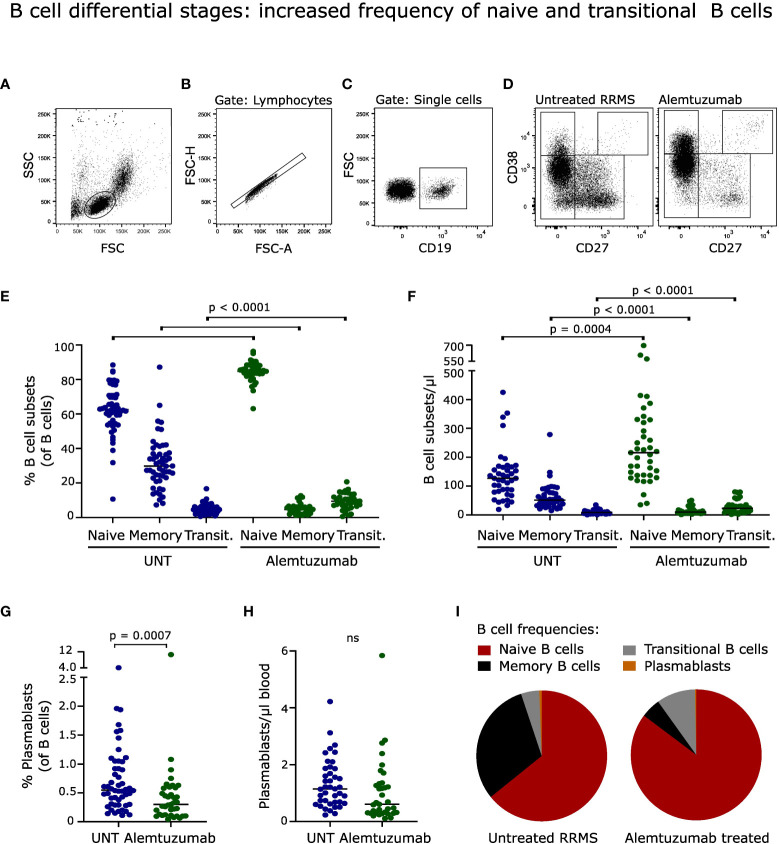
B cell differential stages. **(A-D)** Flow cytometry dot plot example of gating strategy. B cells were defined as CD19^+^ cells and further divided into naïve (CD27^-^CD38^+/-^), transitional (CD27^-^CD38^++^), memory (CD27^+^CD38^+/-^) B cells and plasmablasts (CD27^++^CD38^++^). Frequency and absolute numbers of B cell subtypes **(E, F)** and **(G, H)** plasmablasts in untreated (UNT) and alemtuzumab-treated patients. **(I)** Frequency distribution of B cell subtypes. The median value is shown for all groups analyzed. ns, non-significant.

**Figure 3:**
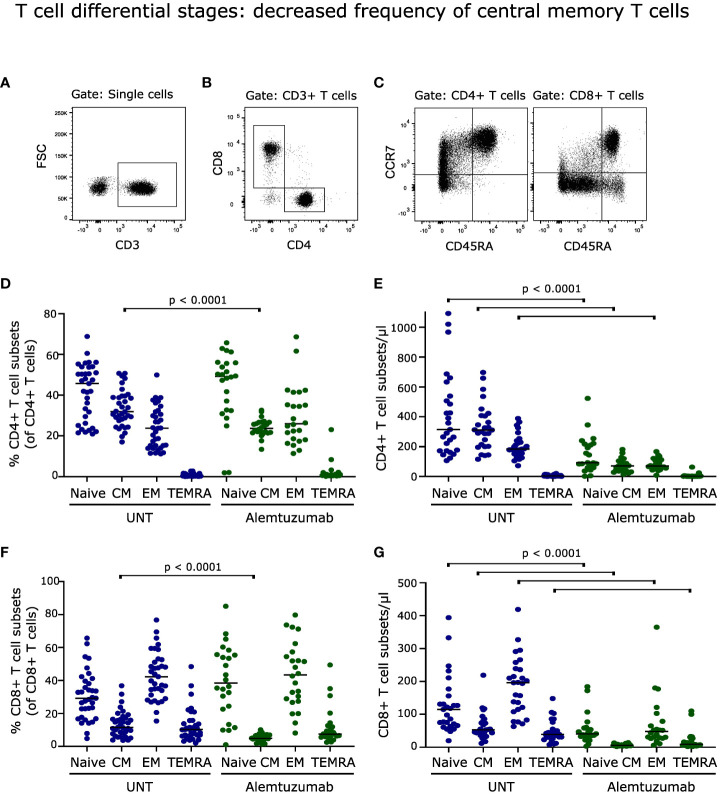
T cell differential stages. **(A–C)** Flow cytometry dot plot example of gating strategy. T cells were defined as CD3^+^ cells, subdivided into CD4^+^ and CD8^+^ cells, and further into naïve (CD45RA^+^CCR7^+^), central memory (CM; CD45RA^-^CCR7^+^), effector memory (EM; CD45RA^-^CCR7^-^) and terminally differentiated (CM; CD45RA^+^CCR7^-^) T cells. Frequency and absolute numbers of CD4^+^ T cell subtypes **(D, E)** and CD8^+^ T cell subtypes **(F, G)** in untreated (UNT) and alemtuzumab-treated patients. The median value is shown for all groups analyzed. ns, non-significant.

The lower absolute lymphocyte count in reconstituted alemtuzumab-treated patients was reflected in a lower count of CD4^+^ and CD8^+^ T cells (p < 0.0001, **Figure 1D**), CD20^+^CD4^+^ and CD20^+^CD8^+^ T cells (p < 0.0001), and NK cells (p = 0.0002) compared to untreated patients. In contrast, we observed a normalized number of B cells (**Figure 1I**), and an increased number of CD56^hi^ NK cells (p < 0.0001, **Figure 1M**). Gating examples of T, B and NK cells are shown in **Figures 2**, **3** and **Supplementary Figure 1**.

### B cell differential stages: increased frequency of naïve and transitional B cells

Despite a normalized number of B cells following two years of alemtuzumab treatment, the distribution of B cell subsets was profoundly changed (**Figure 2I**). The frequency of naïve (CD27^-^CD38^-/+^) and transitional (CD27^-^CD38^++^) CD19^+^ B cells was increased (p < 0.0001); and memory B cells (CD27^+^CD38^-/+^) and plasmablasts (CD27^++^CD38^++^) decreased (p < 0.0001; p = 0.0007) compared to untreated patients, **Figures 2E, G**. A gating example is shown in **Figures 2A–D**.

The same pattern was observed for absolute B cell subset counts with an increased number of naïve (p = 0.0004) and transitional (p <0.0001) B cells, and a decreased number of memory B cells (p < 0.0001), **Figure 2F**. The absolute count of plasmablasts was slightly but nonsignificantly reduced (p = 0.0160), **Figure 2H**.

### T cell differential stages: decreased frequency of central memory T cells

Analyzing the distribution of naïve and memory T cells following two years of alemtuzumab treatment showed a decreased frequency of central memory (CCR7^+^CD45RA^-^) CD4^+^ and CD8^+^ T cells (p < 0.0001); with no significant changes in either percent naïve (CCR7^+^CD45RA^+^), effector memory (CCR7^-^CD45RA^-^) or terminally differentiated (CCR7^-^CD45RA^+^) T cell populations, **Figures 3D, F**. A gating example is shown in **Figures 3A–C**.

Although only the percentage of central memory T cells was affected by alemtuzumab treatment, the reduced total counts of CD4^+^ and CD8^+^ T cells resulted in a lower absolute number of naïve, central memory and effector memory CD4^+^ T cells (p < 0.0001, **Figure 3E**) and of naïve, central memory, effector memory and terminally differentiated CD8^+^ T cells (p < 0.0001, **Figure 3G**).

### Regulatory and effector T cells: increased control of T effector cells

**Figure 4:**
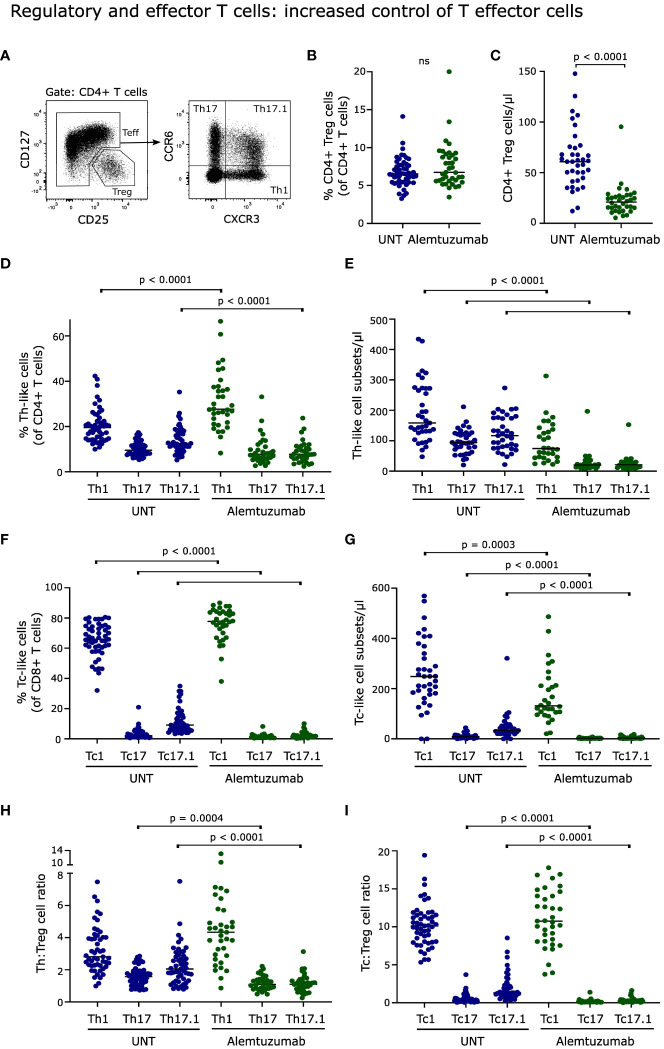
Regulatory and effector T cells. **(A)** Flow cytometry dot plot example of gating strategy. Effector T cells (Teff) and regulatory T cells (Treg) can be defined according to their expression of CD25 and CD127. Teff cells can be further subdivided into functionally different subsets; CXCR3^+^CCR6^-^ (Th1/Tc1-like), CXCR3^-^CCR6^+^ (Th17/Tc17-like), CXCR3^+^CCR6^+^ (Th17.1/Tc17.1-like). Frequency and absolute numbers of Treg cells **(B, C)**, Th-subsets **(D, E)**, Tc-subsets **(F, G)** in untreated (UNT) and alemtuzumab-treated patients. Th : Treg cell ratios **(H)** and Tc : Treg cell ratios **(I)** of untreated (UNT) and alemtuzumab-treated patients. The median value is shown for all groups analyzed.

Regulatory T (Treg) cells act to suppress immune responses to maintain self-tolerance. The percentage of CD4^+^CD127^-^CD25^++^ Treg cells after two years of reconstitution was comparable to the frequency in untreated patients (**Figure 4B**); but due to the lower number of repopulated CD4^+^ T cells the absolute number of Treg cells was decreased (p < 0.0001, **Figure 4C**). A gating example is shown in **Figure 4A**.

Effector T (Teff) cells can be subdivided into functionally different subsets according to their expression of CXCR3 and CCR6. Following two years of alemtuzumab treatment there was an increased frequency of CD4^+^ CXCR3^+^CCR6^-^ (Th1-like; p < 0.0001) and CD8^+^ CXCR3^+^CCR6^-^ (Tc1-like; p < 0.0001) T cells compared to untreated patients, **Figures 4D, F**. Conversely, we found a reduced frequency of CD4^+^ CXCR3^+^CCR6^+^ (Th17.1-like; p < 0.0001), CD8^+^ CXCR3^-^CCR6^+^ (Tc17-like; p < 0.0001) and CD8^+^ CXCR3^+^CCR6^+^ (Tc17.1-like; p < 0.0001) T cells, **Figures 4D, F**. As a result of the lower number of repopulated CD4^+^ and CD8^+^ T cells, all six CXCR3 CCR6 populations were significantly reduced in absolute numbers, **Figures 4E, G**.

A skewing towards a lower T effector:Treg cell ratio towards the tolerogenic Treg component, signifies a more immunosuppressive environment with potential for increased control of T effector cell activation. After two years of alemtuzumab treatment, we observed a significantly reduced ratio between Th17, Th17.1, Tc17 and Tc17.1-like cells and Treg cells (p < 0.0001), **Figures 4H, I**. No significant changes was observed in the Th1:Treg or Tc1:Treg cell ratios, despite an increased frequency of both Th1 and Tc1 cells. CD20^+^ T cells are effector T cells of a Th1/Tc1-like phenotype with a proposed proinflammatory role in RRMS (14–16). In this study, we observed a reduced ratio of both CD4^+^CD20^+^ T cells and CD8^+^CD20^+^ T cells and Treg cells (p < 0.0001) following two years of alemtuzumab therapy, implying an increased control of this subset along with the Th17/Th17.1/Tc17/Tc17.1-like subsets.

### T cell exhaustion following alemtuzumab treatment

**Figure 5:**
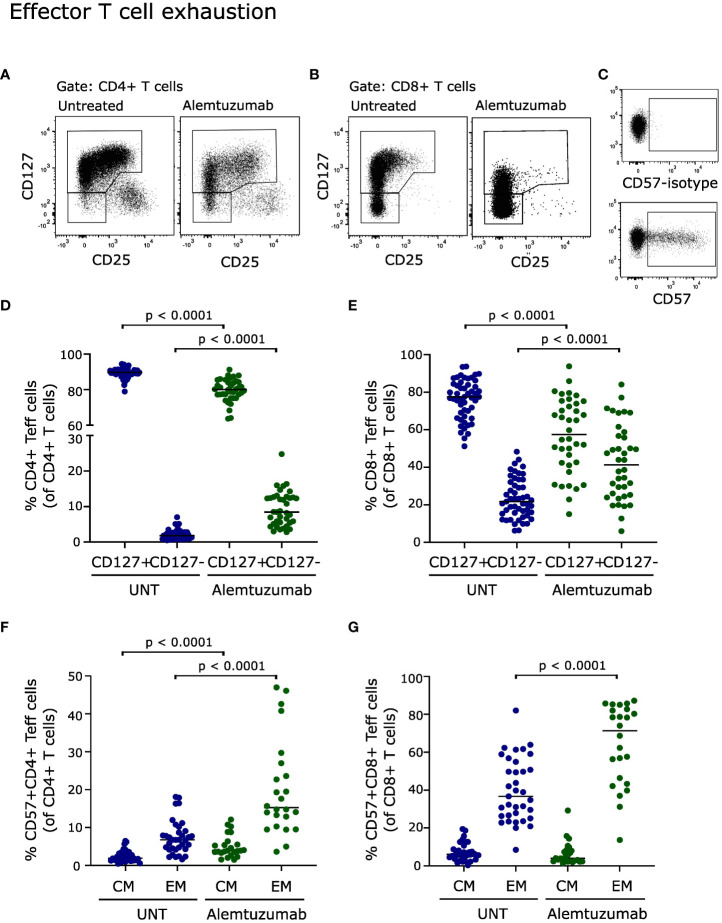
Effector T cell exhaustion. Flow cytometry dot plot examples of gating strategy. Exhausted T cells were defined as CD127^-^CD4^+^ effector T cells **(A)**, CD127^-^CD8^+^ effector T cells **(B)** and CD57^+^ **(C)**. Frequency of CD127^+^ and CD127^-^ CD4^+^ **(D)** and CD8^+^ **(E)** effector T cells of untreated (UNT) and alemtuzumab-treated patients. Frequency of CD57^+^ CD4^+^ **(F)** and CD8^+^ **(G)** central memory (CM) and effector memory (EM) T cells of untreated (UNT) and alemtuzumab-treated patients. The median value is shown for all groups analyzed.

Conditions that cause prolonged activation of the immune system, including repopulation following immune-depletion therapy, may induce a state of T cell dysfunction known as T cell exhaustion (17). To analyze T cell exhaustion, we measured the loss of CD127 as a marker of exhaustion on effector T cells (17, 18). This showed a significant increase in the frequency of CD127^-^ CD4^+^ and CD8^+^ T cells (p < 0.0001, **Figures 5D, E**). A gating example is shown in **Figures 5A, B**.

T cell exhaustion is also associated with expression of CD57 (19, 20). Analyzing CD57 expression on repopulated T cells from alemtuzumab treated patients showed a strong increase in CD57^+^ CD4^+^ central memory and effector memory T cells (p < 0.0001, **Figure 5F**) and in CD57^+^ CD8^+^ effector memory T cells (p < 0.0001, **Figure 5G**). Furthermore, a correlation analysis showed a direct correlation between CD57 upregulation and loss of CD127 on CD8^+^ T cells (p = 0.0006, r_s_ = 0.7). A gating example is shown in **Figure 5C**.

### Follicular T cells: high PD-1 expression and decreased B cell control

**Figure 6:**
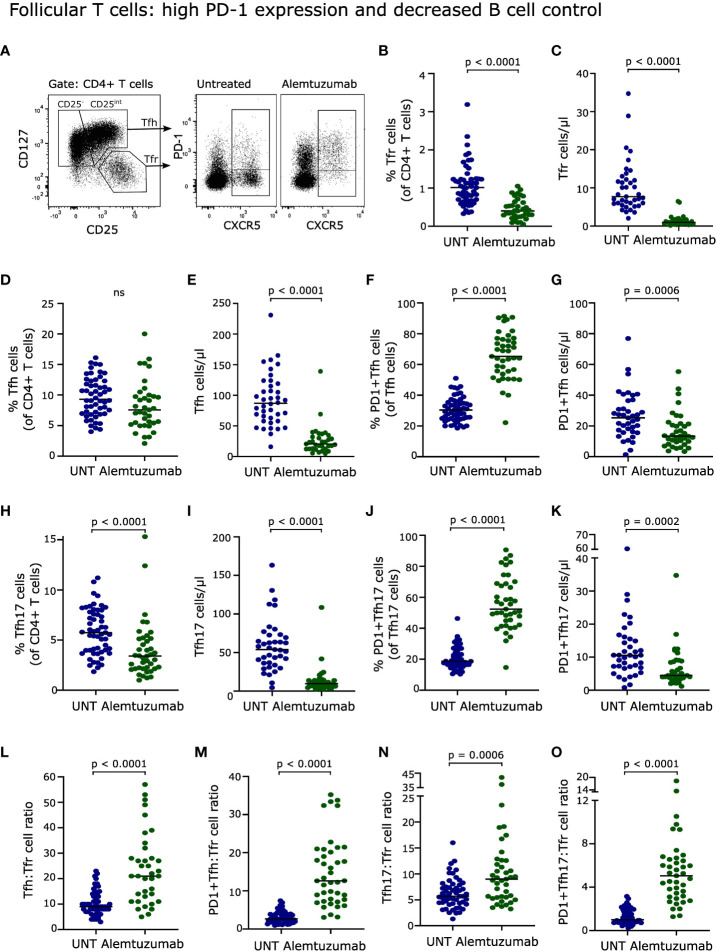
Follicular T cells. **(A)** Flow cytometry dot plot example of gating strategy. Tfh cells were defined as CD4^+^CD25^-/int^ CD127^+^CXCR5^+^PD-1^+/-^ T cells, Tfh17 cells as CD4^+^CD25^int^CD127^+^CXCR5^+^PD-1^+/-^ T cells, and Tfr cells as CD4^+^CD25^hi^CD127^-^CXCR5^+^ T cells. Frequency and absolute numbers of Tfr cells **(B, C)**, Tfh cells **(D, E)**, PD-1^+^Tfh cells **(F, G)**, Tfh17 cells **(H, I)** and PD-1^+^Tfh17 cells **(J, K)** of untreated (UNT) and alemtuzumab-treated patients. Tfh : Tfr cell ratios **(L)**, PD-1^+^Tfh : Tfr cell ratios **(M)**, Tfh17:Tfr cell ratios **(N)** and PD-1^+^Tfh17:Tfr cell ratios **(O)** of untreated (UNT) and alemtuzumab-treated patients. The median value is shown for all groups analyzed.

Follicular helper T (Tfh) cells, defined as CD4^+^CD127^+^CXCR5^+^PD1^+/-^ T cells (21), are important for B cell activation, antibody production and survival (22, 23). The Tfh subpopulation Tfh17 is of particular interest as it promotes activation of naïve B cells (24). CD25^int^ Tfh cells has previously been shown to represent CXCR3^-^CCR6^+^ Tfh17 cells (21). In this study, we therefore defined Tfh17 cells as CD25^int^ Tfh cells, see **Figure 6A**. PD-1 is upregulated on the surface of Tfh cells upon cognate antigen activation (25). As T cells after two years of alemtuzumab therapy is characterized by extensive activation, PD-1 expression analysis was included. We observed that the frequency of Tfh cells was slowly normalizing (**Figure 6D**) after two years of treatment, in contrast to Tfh17 cells that were still suppressed (p < 0.0001, **Figure 6H**). We also found, that most Tfh and Tfh17 cells expressed PD-1 in contrast to what was observed in untreated patients (p < 0.0001, **Figures 6F, J**). Due to the lower absolute count of CD4^+^ T cells in treated patients all four Tfh-populations were reduced in numbers (**Figures 6E, G, I, K**).

The regulatory counterpart of Tfh cells, follicular regulatory T (Tfr) cells, defined as CD4^+^CD127^-^CD25^++^CXCR5^+^ T cells (21), have the potential for immune regulation of Tfh cells and hence of B cell activation including suppression of high-affinity antibody production (22, 23). Following two years of alemtuzumab treatment, Tfr cells were also strongly reduced both in percentage and absolute numbers (p < 0.0001, **Figures 6B, C**).

The capacity of Tfr cells to control the activity of Tfh and hence of B cells can be evaluated by the Tfh : Tfr cell ratio. This showed a significant increase in the ratio between Tfh, PD-1^+^Tfh, Tfh17, PD-1^+^Tfh17 and Tfr cells, indicating a decreased control of all four Tfh-populations (p < 0.0001, p = 0.0006 for Tfh17 cells, **Figures 6L–O**).

### Immune activation and secondary autoimmune disease

T cell exhaustion has been suggested as a marker of alemtuzumab-treated patients who develop secondary autoimmunity (26). Investigating an association between T cell exhaustion and development of thyroiditis showed no correlation between development of thyroid autoimmunity and either percent or absolute count of exhausted CD4^+^ or CD8^+^ T cells defined by loss of CD127 or gain of CD57. Furthermore, the extensive activation of Tfh and Tfh17 cells observed in this study as an upregulation of PD-1 was not associated with development of thyroiditis (**Table 1**).

**Table 1:** Immune cell markers, disease activity and secondary autoimmune disease.

Immune markers	Thyroiditis (9/40); *p*	NEDA (13/40); *p*	Attacks (21/40); *p*	MRI activity (12/40); *p*
Exhausted T cells:				
%CD4^+^CD127^-^ (of CD4^+^ T cells)	0.43	0.71	0.34	0.41
%CD8^+^CD127^-^ (of CD8^+^ T cells)	0.06	0.36	0.97	0.15
CD4^+^CD127^-^ cell count	0.34	0.29	0.27	0.25
CD8^+^CD127^-^ cell count	0.27	0.69	0.95	0.74
%CD4^+^CD57^+^ EM (of CD4^+^ EM T cells)	0.13	0.49	0.74	0.65
%CD8^+^CD57^+^ EM (of CD8^+^ EM T cells)	0.16	0.16	0.009	0.51
CD4^+^CD57^+^ cell count	0.09	0.75	0.84	0.57
CD8^+^CD57^+^ cell count	0.21	0.35	0.34	0.85
Tfh cells:				
%Tfh (of CD4^+^ T cells)	0.61	0.83	0.62	0.89
%Tfh17 (of CD4^+^ T cells)	0.64	0.05	0.22	0.30
%PD1^+^Tfh (of Tfh cells)	0.13	0.01	0.31	0.67
%PD1^+^Tfh17 (of Tfh17 cells)	0.09	0.0076	0.22	0.89
Tfh cell count	0.31	0.29	0.60	0.89
Tfh17 cell count	0.22	0.15	0.99	0.64
PD1^+^Tfh cell count	0.37	0.77	0.65	0.69
PD1^+^Tfh17 cell count	0.11	0.53	0.95	0.98
B cells:				
% B (of lymphocytes)	0.63	0.84	0.70	0.65
B cell count	0.65	0.49	0.16	0.79
B cell control:				
Tfh : Tfr cell ratio	0.26	0.90	0.59	0.85
Tfh17:Tfr cell ratio	0.71	0.26	0.82	0.40
PD1^+^Tfh : Tfr cell ratio	0.60	0.40	0.68	0.81
PD1^+^Tfh17:Tfr cell ratio	0.77	0.88	0.99	0.51
*CD20^+^ T cells:				
%CD4^+^CD20^+^ (of CD4^+^ T cells)	0.93	0.0009	0.02	0.69
%CD8^+^CD20^+^ (of CD8^+^ T cells)	0.59	0.70	0.69	0.82
CD4^+^CD20^+^ cell count	0.83	0.030	0.09	0.19
CD8^+^CD20^+^ cell count	0.41	0.86	0.62	0.57
*CD20^+^ T cell control:				
CD4^+^CD20^+^ T:Treg ratio	0.15	0.0003	0.04	0.44
CD8^+^CD20^+^ T:Treg ratio	0.57	0.72	0.72	0.70

*Without correction for the mononuclear CD20-antibody used for analysis.

Thyroid autoimmunity is considered to be B cell driven and anti-thyroid antibody mediated (27); however, we did not find a correlation between development of thyroiditis and B cells or plasmablasts, **Table 1**. The balance between Tfh and Tfr cells is important in maintaining immune tolerance primarily by controlling the activity of B cells (22). However, analyzing a possible association between the Tfh : Tfr cell ratio and development of thyroiditis also showed no correlation (**Table 1**).

Proinflammatory CD20^+^ T cells are likely implicated in various autoimmune diseases (28). Analyzing a possible association between CD20^+^ T cells and development of thyroiditis showed no association between thyroid autoimmunity and either percentage or absolute counts of CD4^+^CD20^+^ or CD8^+^CD20^+^ T cells, nor of CD20^+^ T cell:Treg ratios (**Table 1**).

### Immune activation and disease activity

In order to analyze the relationship between immune cell subsets and disease activity, we compared values in patients with NEDA in year two and three after initiation of treatment. In addition, we assessed any relationship with the number of relapses during the three years of treatment or the development of confirmed disability worsening (CDW).

A previous study indicated an association between IL-17 producing CD4^+^ T cells and patients experiencing a relapse following 18 months of alemtuzumab treatment (29). We therefore analyzed a possible association between NEDA, relapses and CDW and the prevalence of Th-subsets including IL-17 producing Th17 and Th17.1-like cell, and found no correlations (**Table 1**). CD20^+^ T cells have also been associated with disease activity in patients with RRMS (14–16). We found some relationship between CD20^+^ T cells and disease activity in the entire material (**Table 1**); however, we found no significant relationship in multivariable analysis with correction for the monoclonal antibody used for analysis (data not shown). Also, in the larger subgroup of 25 patients studied with the BV421-conjugated anti-CD20 antibody, we found no significant relationship between CD20^+^ T cells and NEDA or relapses. Neither did we find any relationship between any of the blood cell subsets and the total number relapses during the three years of treatment or the development of CDW (data not shown).

## Discussion

Alemtuzumab has proven to be a highly effective therapy in suppressing neuroinflammation and preventing relapses in RRMS (2, 3, 30). After a first and a second course of alemtuzumab administration circulating lymphocytes are profoundly depleted, followed by a gradual repopulation of lymphocytes that do not reach baseline levels for several years (7, 8). The long-term protective effect of alemtuzumab likely consist in the reprogramming of immune cell subsets essential for permanent remission to a greater extent than the lower lymphocyte count. The composition of the reconstituted lymphocyte population therefore has been investigated with great interest. In contrast to many of the previous explorative studies, our study is based exclusively on the use of freshly isolated cells (< 1 h of sampling) to avoid any bias in relation to cryopreservation as has been observed for certain CD4^+^ and CD8^+^ T cell populations (31). In addition, we have performed a thorough immune cell phenotyping of a large number of lymphocyte subsets, enabling a coherent overview of the lymphocyte compartment following reconstitution. Besides analysis of cell frequencies, we have also included a calculation of absolute cell counts. As alemtuzumab is a cell depleting therapy, studies leaving out cell count measurements miss important information. A limitation in our study is the lack of baseline samples. Instead, we compared patients treated for two years with a group of age and sex matched treatment-naïve patients with RRMS; however, as the alemtuzumab-induced effect on lymphocytes are profound the introduced confounding is less noteworthy.

Consistent with previous observations (7, 8, 32–34), phenotyping of peripheral lymphocyte subsets showed an alemtuzumab-induced redistribution of T, B and NK cells characterized by a decreased frequency in CD4^+^ T cells and an increased frequency of B cells and NK cells; the latter explained by a large increase in the percentage of regulatory CD56^hi^ NK cells. In absolute cell numbers, we observed that CD4^+^ and CD8^+^ T cells were decreased, B cells were normalized and CD56^hi^ NK cells increased despite an overall lower number of NK cells. Within the B cell compartment, we found primarily naive and transitional B cells after two years of alemtuzumab therapy, as also reported by others (7, 9). B cells from alemtuzumab treated patients have been shown to have an increased capacity to produce the anti-inflammatory cytokine IL-10 and functionally to inhibit the proliferation of CD4^+^ effector T cells (35). IL-10 producing B cells, termed regulatory B (Breg) cells, do not have unique plasma membrane markers in humans; instead, they are of a general activated B cell phenotype (36). An earlier study showed that B cells from alemtuzumab-treated patients produced IgM and not IgG (37), confirming the antigen-naïve B cell phenotype of reconstituted patients. We propose that the IL-10 producing capacity lies within the transitional B cell population, as we previously have shown transitional B cells (CD19^+^CD27^-^CD38^++^) to produce IL-10; and additionally that they are the main B cell producers of TGFβ, another cytokine with anti-inflammatory potential (38). The prolonged reduction in memory B cells and the increase in B cells with regulatory capacity has therefore been suggested as a contributing factor of the long-term efficacy of alemtuzumab. Despite this, we did not find an association between transitional or other B cell subtypes with NEDA, attacks or MR activity.

Besides a suggested role of B cells in the long-lasting effect of alemtuzumab in patients with RRMS, a shift in the balance between proinflammatory and regulatory T cells has been proposed (29). Patients experiencing an attack following 18 months of alemtuzumab treatment have an increased prevalence of CD4^+^ T cells producing the proinflammatory cytokine IL-17 (29). The IL-17 and IFN-γ double-producing Th-subset Th17.1 has previously been associated with clinical disease activity in patients with RRMS (39), and we therefore hypothesized that a low frequency of Th17.1 cells were associated with response to treatment. Indeed, the only Th-effector cell subset to be reduced both in frequency and absolute counts was Th17.1 cells. Furthermore, we observed that Th17.1 cells (along with Th17 cells) were under increased control of regulatory T cells following two years of alemtuzumab treatment, seen as a markedly reduced Th17.1:Treg cell ratio. Earlier studies have shown, that Treg cell function was intact following alemtuzumab therapy (10, 33), confirming the impact of a reduced Th17.1:Treg cell ratio on Th17.1 cell activity. Analyzing a possible association between Th17.1 cells and Th17.1:Treg cell ratio and disease activity defined as NEDA, attacks or MR activity did, however, not show any correlation.

Another proinflammatory T effector cell subset that has been investigated recently is the CD20 positive T cell subset. The frequency of CD20^+^ T cells is increased in the blood, enriched in the CSF of patients with RRMS, are highly reactive to myelin antigens, are great producers of IFNγ, TNFα and GM-CSF, and are likely involved in the nervous tissue damage observed in MS (14, 16, 40). We have previously shown that alemtuzumab effectively reduces the prevalence of CD20^+^ T cells both in blood and in cerebrospinal fluid in a smaller cohort of patients (14), a finding confirmed in the current study both applicable to CD4^+^ and CD8^+^ CD20^+^ T cells. A previous study found a reduction in IFNγ-production by CD4^+^ T cells after two years of alemtuzumab treatment that was likely due of a shift in the distribution of different CD4^+^ T cell subtypes (41). Our data suggests that the IFNγ-reduction observed may partly be explained by the long-term depletion of CD4^+^CD20^+^ T cells. We did not, find evidence that the effects on CD20^+^ T cells are associated with disease activity in patients treated with alemtuzumab, but this may be confounded by the use of two different anti-CD20 monoclonal antibodies during the conduct of the study. This was mandated by a necessary upgrading of our flow cytometry equipment.

Analyzing the reconstituted T cell population after two years of alemtuzumab therapy also displayed a pronounced degree of T cell activation on the path to exhaustion. T cell exhaustion is a result of prolonged immune activation and is characterized by a progressive loss of function (17). In our study, T cell exhaustion was defined by a cellular loss of CD127 (IL-7Rα); a receptor important for IL-7-dependent homeostatic survival of memory T cells (42). Loss of CD127 expression is associated with chronic immune activation (43); and in our study we found an expansion of both CD127^-^ CD4^+^ and CD8^+^ T cells, an observation most pronounced for CD8^+^ T cells where almost half the population had downregulated CD127. CD127^-^CD8^+^ T cells are a population enriched in both activated effector/memory T cells and terminally differentiated T cells (43). Exhausted CD8^+^ T cells most likely arise from functional memory T cells rather than from terminally differentiated cells (44). If relieved from chronic activation before being irreversibly committed to exhausted cells, they can revert to functional memory CD8^+^ T cells (44). As we did not perform functional studies on the CD127^-^ T cells we cannot assess their exhaustion state; however, it has been previously shown that CD127^-^CD8^+^ T cells have less antiapoptotic molecules and gradually lose their potential as functional memory T cells and die (44–46). The observation of a markedly increased population of CD127^-^CD8^+^ T cells in the current study may reflect a loss of responsiveness to IL-7 and failure to reconvert to functional memory T cells, hence representing a highly compromised immune cell subset. To strengthen these data, we also measured CD57 expression on memory T cells of alemtuzumab-treated patients. CD57 expression on CD4^+^ and CD8^+^ T cells is associated with proliferation incompetence and replicative senescence and like CD127 expression is associated with chronic infection (20). In our study, we found a large increase in CD4^+^ and CD8^+^ effector memory T cells (CD45RA^-^CCR7^-^) expressing CD57, and furthermore that CD57^+^CD8^+^ and CD127^-^CD8^+^ T cells were directly correlated.

Tfh cells of patients treated for two years with alemtuzumab is likewise characterized by a high degree of activation as most Tfh cells expressed PD-1. PD-1 is an activation marker upregulated on the surface of Tfh cells following their first antigen encounter (25) and has been proposed as a marker of CD4^+^ T cell exhaustion (47); however, an involvement of PD-1 in Tfh cell exhaustion is unknown. The prime function of Tfh cells is to promote B cell proliferation and differentiation to antibody-producing plasma cells to encourage and regulate humoral responses (23). A subpopulation of Tfh cells, Tfh17 cells, has been described as efficient inducers of naïve B cell activation and production of IgG in contrast to Tfh1 cells that lacks the capacity to help naïve B cells (24). Considering the increase in naïve B cells following alemtuzumab therapi and the risk of developing autoimmune IgG antibodies, we focused our analysis on the B cell activation potential of Tfh17 cells. This showed a large increase in the ratio between Tfh17 cells and PD-1^+^ Tfh17 cells and their regulatory counterpart Tfr cells, reflecting a decreased control of Tfh-activity and hence of B cell activation. Along with the high level of naïve B cells, the observed increased Tfh : Tfr cell ratio likely provides part of the explanation as to why patients reconstituted from alemtuzumab therapy (> 6 month from last treatment) retain the ability to mount a humoral immune response against vaccines based on T cell-dependent antigens despite reduced CD4^+^ T cell counts (48).

Besides the limitations of the study discussed above, a limitation to highlight is identifying cell subsets based only on cell surface markers leaving out functional studies. Also, it would have been interesting to include multiple time points following alemtuzumab administration to gain insight into reconstitution kinetics; however, these approaches were outside the scope of the study.

During reconstitution of the lymphocyte repertoire following alemtuzumab treatment, patients are at high risk of developing secondary autoimmunity (27). Within the three years of our study 22% developed thyroiditis. Thyroiditis is a group of disorders considered to be B cell-driven and anti-thyroid antibody mediated (27). The etiology of these autoantibodies is unknown, but suppression of regulatory immune cells may be a contributing cause. In our study we found that regulatory Tfr cells, whose function is to control B cell activation and production of high affinity antibodies (21, 22), was severely reduced both in frequency and absolute number, impacting the balance (ratio) between Tfh and Tfr cells in favor of Tfh cells and hence B cell activity. Despite this, we did not find an association between the Tfh : Tfr cell ratios and development of thyroiditis in the patients. Analyzing a larger patient cohort or a later time point where more patients may develop thyroiditis (there is a 5-year incidence of 40%) (49) would possibly strengthen an association. Anti-thyroid antibodies are often present prior to manifestation of thyroiditis, possibly providing a more sensitive measurement of an early association between thyroiditis and Tfh : Tfr cell ratio; unfortunately, we did not perform this analysis. A lack of regulatory T cell control may also be due to the imbalance in T and B cell reconstitution kinetics, with a delayed T cell repopulation compared to the early and marked B cell hyperrepopulation (9).

In lymphopenic hosts with a reduced thymic output of naïve T cells as observed following alemtuzumab therapy, peripheral T cells proliferate in response to self-antigens (homeostatic expansion) (50) leading to chronically activated oligoclonal memory T cells and likely a predisposition to develop autoimmune disease (51). A recent publication investigating the clonality of T cells in reconstituted patients with MS following alemtuzumab treatment reported hyperexpanded T cell clones with a restricted antigen recognition repertoire (26). Also, homeostatic proliferation leading to hyperexpanded T cell clones identified patients developing secondary autoimmunity (26). Hyper expansion and development of antigen-restricted T cell clones may be an indication of present or forthcoming T cell exhaustion; however, we did not find an association between patients with an exhausted T cell profile and development of thyroiditis. Development of secondary autoimmunity following alemtuzumab therapy likely reflects a coincidence of several unfortunate factors including a skewed Tfh : Tfr cell ratio, homeostatic proliferation of T cells, a delayed Treg response and exhausted T cells. Also, a hereditary susceptibility to development of secondary autoimmunity may be involved. Secondary autoimmunity only occurs in predisposed individuals like patients with MS, not in patients receiving alemtuzumab for the treatments of cancer (52).

Altogether, our data suggests that skewing of the reconstituted immune response towards a more immunosuppressive environment of MS-associated proinflammatory T cell responses may contribute to the long-lasting effect of alemtuzumab therapy.

## Data availability statement

The raw data supporting the conclusions of this article will be made available by the authors, without undue reservation.

## Ethics statement

The studies involving humans were approved by The regional scientific ethics committee (protocol number H-16047666). The studies were conducted in accordance with the local legislation and institutional requirements. The participants provided their written informed consent to participate in this study.

## Author contributions

MvE performed most of the experiments, collected the study material, analyzed data, wrote the manuscript, and contributed to the design of the study. HC collected the study material, analyzed data, and contributed to the design of the study. RHH and SB collected the study material and performed some of the experiments. FS conceptualized the research, collected the study material, directed the study, and contributed to analysis of data. All authors revised the manuscript and approved the final version for publication. All authors contributed to the article and approved the submitted version.

## Supplementary material

Supplementary Figure 1 NK cells and CD20^+^ T cells. Flow cytometry dot plot example of FSC-SSC gating of lymphocytes **(A)** followed by gating of single cells **(B)**. **(C)** CD56^int^ NK cells were defined as CD16^+^CD56^int^ and CD56^hi^ NK cells as CD16^-/int^ CD56^hi^ single lymphocytes. **(D)** CD20^+^ T cells were defined as CD3^+^CD20^int^ single lymphocytes.
