# Supplementary figures and images for: Immune reconstitution following alemtuzumab therapy is characterized by exhausted T cells, increased regulatory control of proinflammatory T cells and reduced B cell control
Source: Front Immunol. 2023 Sep 6;14:1249201. doi: 10.3389/fimmu.2023.1249201 (PMC10512074; doi:10.3389/fimmu.2023.1249201)

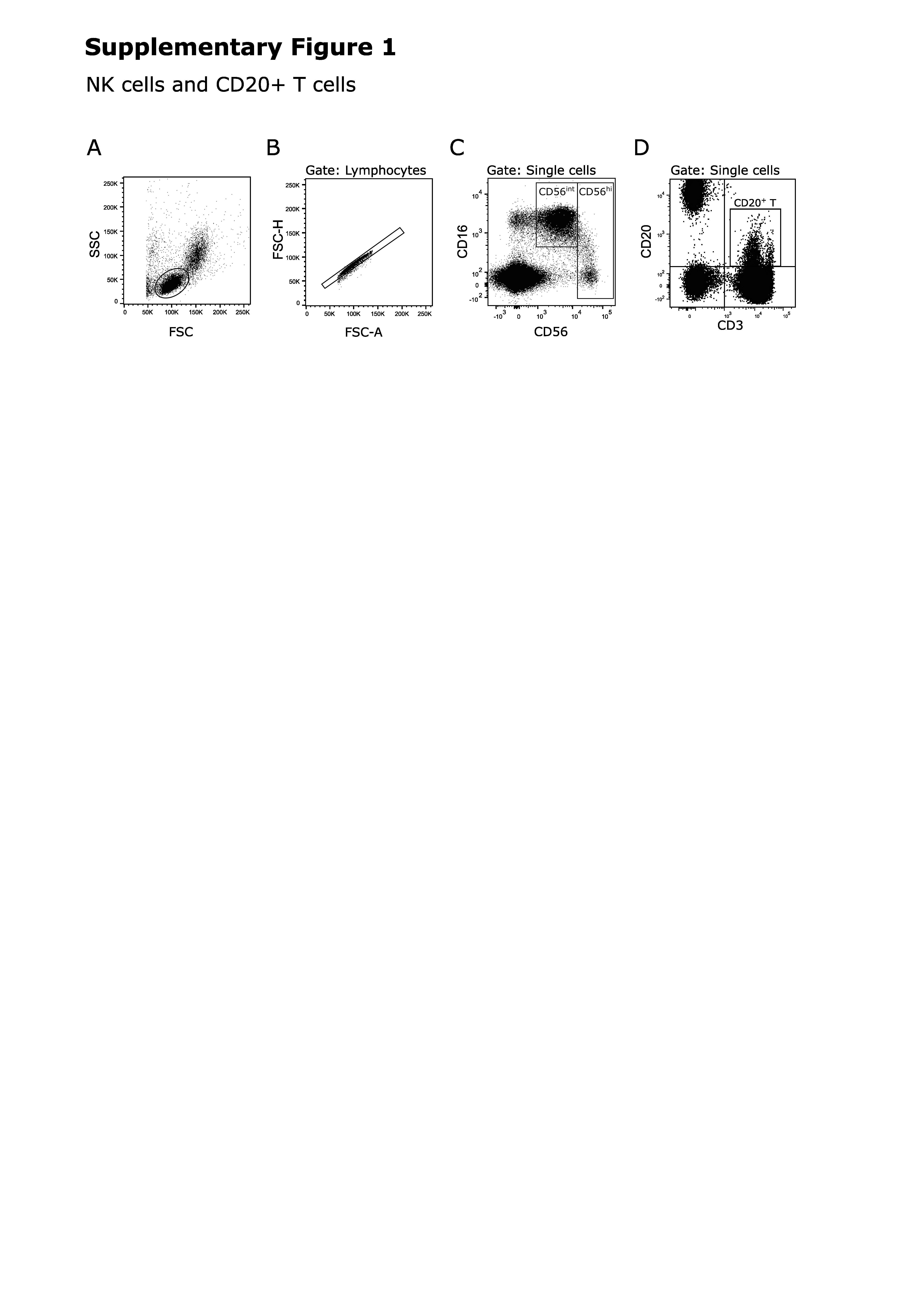

Supplement: Supplementary Figure 1 — NK cells and CD20+ T cells. Flow cytometry dot plot example of FSC-SSC gating of lymphocytes (A) followed by gating of single cells (B). (C) CD56int NK cells were defined as CD16+CD56int and CD56hi NK cells as CD16-/int CD56hi single lymphocytes. (D) CD20+ T cells were defined as CD3+CD20int single lymphocytes. [file Image_1.jpeg]
